# From natural dye to herbal medicine: a systematic review of chemical constituents, pharmacological effects and clinical applications of indigo naturalis

**DOI:** 10.1186/s13020-020-00406-x

**Published:** 2020-12-14

**Authors:** Yang Qi-yue, Zhang Ting, He Ya-nan, Huang Sheng-jie, Deng Xuan, Han Li, Xie Chun-guang

**Affiliations:** 1 Hospital of Chengdu University of Traditional Chinese Medicine, No. 39 Shierqiao Road, Chengdu, 610075 People’s Republic of China; 2 School of Pharmacy, Chengdu University of Traditional Chinese Medicine, Chengdu, People’s Republic of China; 3 Chengdu University of Traditional Chinese Medicine, No. 1188 Liutai Avenue, Chengdu, 611137 China

**Keywords:** Indigo naturalis, Indole alkaloids, Anti-inflammation, Leukemia, Psoriasis, Ulcerative colitis

## Abstract

**Background:**

Indigo naturalis is a blue dye in ancient, as well as an extensive used traditional Chinese medicine. It has a wide spectrum of pharmacological properties and can be used to treat numerous ailments such as leukemia, psoriasis, and ulcerative colitis. This article aims to expand our understanding of indigo naturalis in terms of its chemical constituents, pharmacological action and clinical applications.

**Methods:**

We searched PubMed, web of science, CNKI, Google academic, Elsevier and other databases with the key words of “Indigo naturalis”, and reviewed and sorted out the modern research of indigo naturalis based on our research results.

**Results:**

We outlined the traditional manufacturing process, chemical composition and quality control of indigo naturalis, systematically reviewed traditional applictions, pharmacological activities and mechanism of indigo naturalis, and summarized its clinical trials about treatment of psoriasis, leukemia and ulcerative colitis.

**Conclusions:**

Indigo naturalis has a variety of pharmacological activities, such as anti-inflammatory, antioxidant, antibacterial, antiviral, immunomodulatory and so on. It has very good clinical effect on psoriasis, leukemia and ulcerative colitis. However, it should be noted that long-term use of indigo naturalis may produce some reversible adverse reactions. In summarize, indigo naturalis is an extremely important drug with great value and potential.
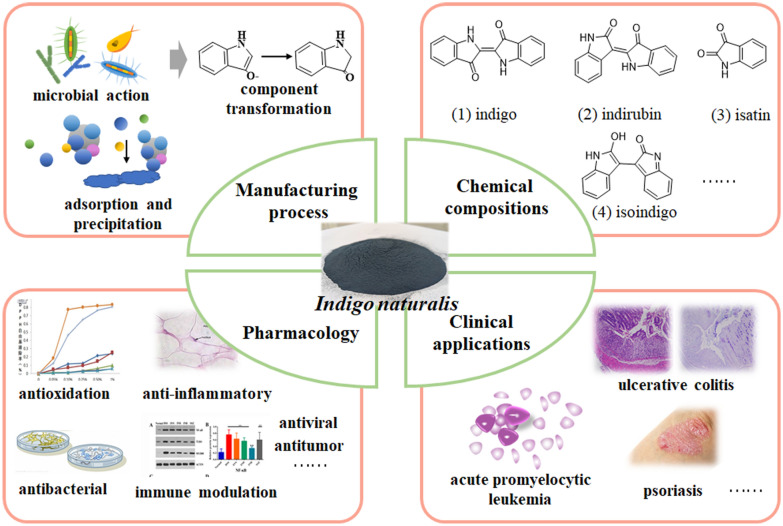

## Introduction

Indigo naturalis is a dried powder or paste processed from the stems and leaves of *Baphicacanthus cusia* (Nees) Bremek. (family Acanthaceae), *Polygonum tinctorium* Ait. (family Polygonaceae) and *Isatis indigotica* Fort. Initially, indigo naturalis was used as a primitive dye in ancient India and then introduced into China through Persia [1]. In the Tang Dynasty,indigo naturalis is a fashionable cosmetic used for thrush. Subsequently, Chinese doctors found it could reduce fever, detoxify the blood, dissolve ecchymoses, and cool down blood [2]. Further study on the material basis of indigo naturalis showed that it was mainly composed of 10% organic matter and 90% inorganic substance, among which organic matter was the main active substance [3]. In addition, pharmacological effects of indigo naturalis were also studied. Modern researches found that indigo naturalis has anti-inflammatory, antioxidant, antibacterial, immune regulation and other activities [4–6]. In recent years, indigo naturalis has also been found to be used in the treatment of cancer, especially indirubin and tryptanthrin have obvious inhibitory effects on various cancers [7, 8]. The discovery of these bioactivities provided positive support for the clinical applications of indigo naturalis. In this manuscript, we reviewed the manufacturing process, chemical composition, traditional applications, pharmacological mechanisms and clinical applications of indigo naturalis, which can provide comprehensive and detailed references to related researchers and contribute to the further development and clinical applications of indigo naturalis.

## Manufacturing process

**Fig. 1:**
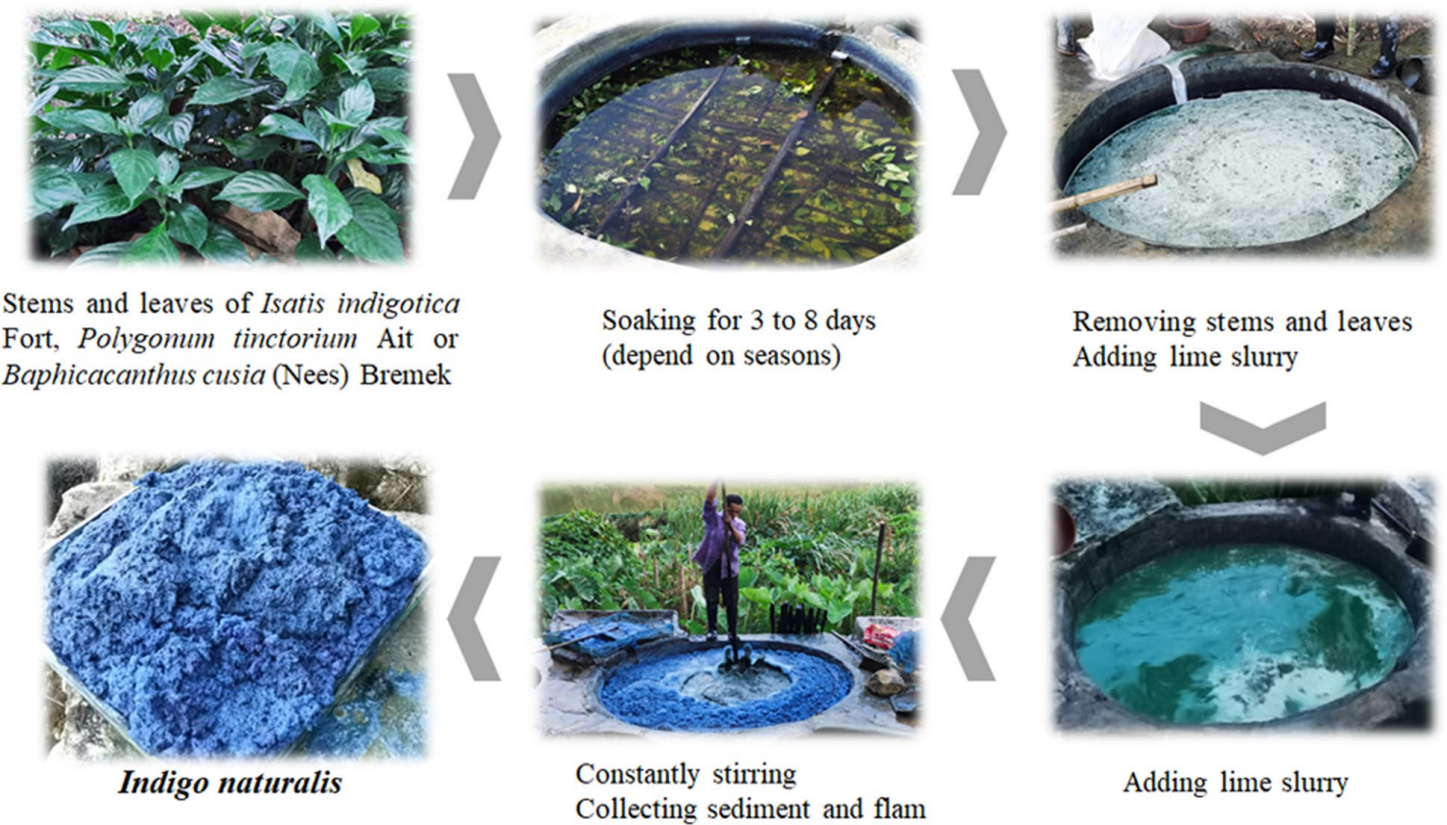
The manufacturing process of Indigo naturalis

**Fig. 2:**
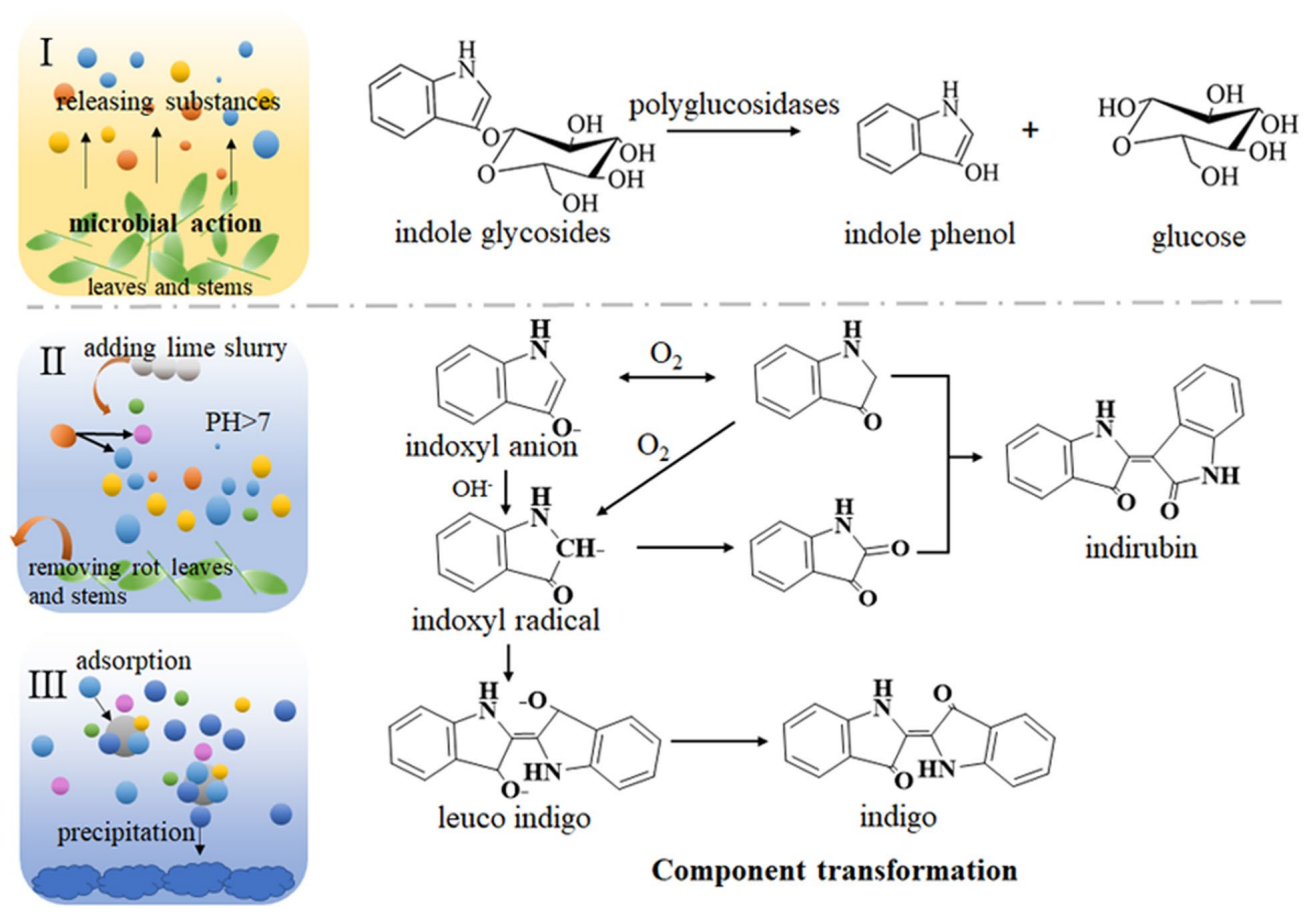
The reaction mechanism of indigo and indirubin.

The conventional manufacturing method includes collecting stems and leaves, soaking, producing coarse indigo, refining, and drying (Fig. 1). The manufacturing process is very interesting, including microbial action, component transformation, adsorption and precipitation (Fig. 2) [6]. Firstly, the stems and leaves from *Baphicacanthus cusia* (Nees) Bremek, *Polygonum tinctorium* Ait or *Isatis indigotica* Fort are soaked in water until rot. Under the action of microorganisms, indole glycosides and endogenous polyglucosidases are released from stems and leaves. In soaking processing, the most important chemical change is that endogenous polyglucosidases hydrolyzes indole glycoside to produce indole phenol, which is the source of indigo and indirubin. Then, stems and leaves are removed and lime slurry is added. Indole phenol combines with oxygen to form indole phenol free radical in an alkaline environment. Some indole phenol radicals condense to form indigo, and some indole phenol radicals are further oxidized to indole ketone and combine with indole phenol to form indirubin. Simultaneously, Ca(OH)_2_ in lime reacts with CO_3_^2−^ in the soaking solution to form the CaCO_3_ nucleus, which provides carriers for adsorbing indigo, indirubin and other compounds. With the continuous deposition of the compound on the surface of CaCO_3_, the gravity of the polymer exceeds the buoyancy, and the solid-liquid separation is realized at the bottom of the water. These polymers are the original indigo naturalis and commercial indigo naturalis is obtained from the further separation, purification and drying of the original indigo naturalis. In fact, the process of soaking and adding lime is not only the conversion of indole phenol, as well as the transformation of other substances. However, the transformation of other substances needs further researches.

## Chemical compositions

Although inorganic substances in indigo naturalis occupy the majority, organic substances are main active components. The organic components can be divided into the following categories [6, 9, 10]. Besides, organic composition ratio of indigo naturalis may not be exactly the same, which depended on the source of stems and leaves.

**Fig. 3:**
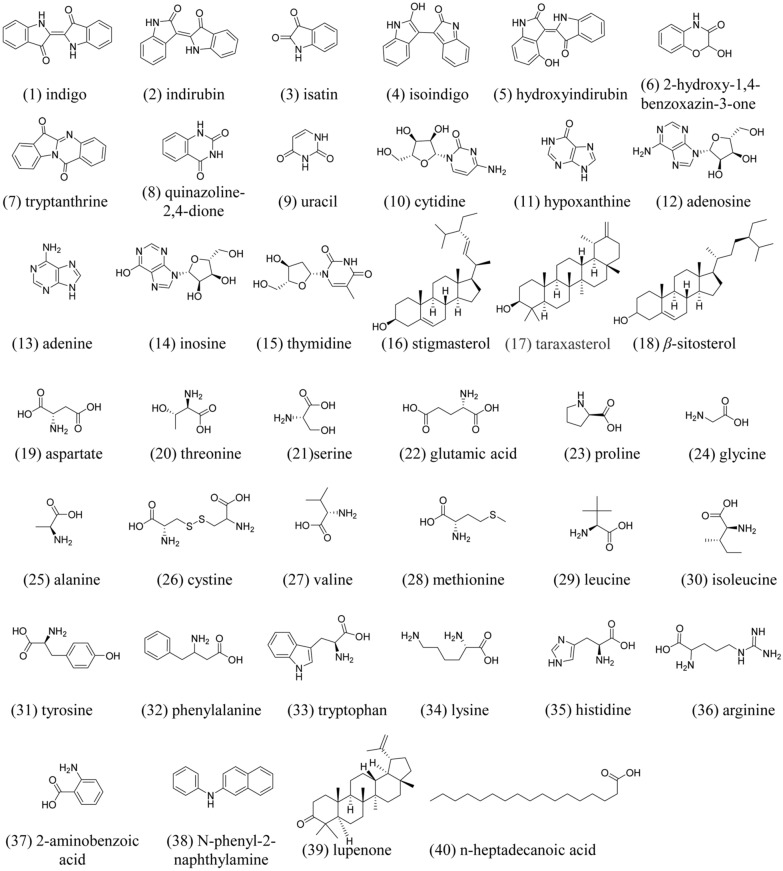
Structural formula of some chemical constituents in indigo naturalis

- Indole alkaloids include indigo (1), indirubin (2), isatin (3), isoindigo (4), hydroxyindirubin (5), 2-hydroxy-1,4-benzoxazin-3-one (6), tryptanthrine (7), quinazoline-2,4-dione (8), etc. (Fig. 3). Indigo, indirubin and isoindigo are two indole alkaloids; moreover, they are isomers.
- Nucleosides include uracil (9), cytidine (10), hypoxanthine (11), adenosine (12), adenine (13), inosine (14) and thymidine (15), etc.
- Sterol compounds include stigmasterol (16), taraxasterol (17), *α*-sitosterol and *β*-sitosterol (18), etc.
- Amino acids include aspartate(19), threonine (20), serine (21), glutamic acid (22), proline (23), glycine (24), alanine (25), cystine (26), valine (27), methionine (28), leucine (29), isoleucine (30), tyrosine (31), phenylalanine (32), tryptophan (33), lysine (34), histidine (35), arginine (36), etc.
- Others include 2-aminobenzoic acid (37), N-phenyl-2-naphthylamine (38), lupenone (39), n-heptadecanoic acid (40), etc.

The inorganic ingredients of indigo naturalis was consist of 70% calcium carbonate, 10% silica dioxide and calcium hydroxide [3, 11]. In addition, it may also include Fe_2_(CO_3_)_3_, Al_2_ (CO_3_)_3_, etc [11]. Calcium carbonate and calcium hydroxide are derived from lime, and silica dioxide and other impurities are derived from soil.

## Quality control

### Character evaluation

Traditional quality evaluation parameters include shape, color, smell, texture, water to test, and fire to test. The best quality is fine powder, blue in color, light and loose in texture, with no sand and stone. In addition, it is able to float in the water, and the water is not blue. When it burns, it gives out a purplish red flame.

### Physical and chemical evaluation

At present, thin layer chromatography (TLC) and high-performance liquid chromatography (HPLC) are the most commonly used quality control methods for indigo naturalis. For instance, the 2015 Chinese pharmacopoeia used TLC to identify indigo and indirubin in samples. The indigo content was more than 2% and that of indirubin was more than 0.13%. Besides TLC and HPLC, there are many other quality control methods for indigo naturalis. Degani et al. [11] describe an analytical procedure based on the silylating agent N,O-bis-(trimethylsilyl) trifluoroacetamide with 1% trimethylchlorosilane for pure molecules of indigo, indirubin and plant extracts of indigo naturalis. Gas Chromatography–mass spectrometry turns out to be an efficient and fast analytical tool for the identification of natural indigo in plants and textile artifacts. Li et al. [9] established a reversed phase HPLC method of simultaneous determination of five nucleosides in indigo naturalis: uracil, cytidine, hypoxanthine, inosine, and thymidine. The method was applied to compare the contents of nucleosides in indigo naturalis from different producing areas and batches. It was found that there were obvious differences in nucleoside content of indigo naturalis from different producing areas or batches. Liao et al. [12] determined the contents of copper, arsenic, cadmium, lead, and mercury in indigo naturalis by inductively coupled plasma mass spectrometry. This method can accurately and rapidly determine the content of heavy metals mentioned above. It is very useful for controlling the quality of indigo naturalis and ensuring its safety.

### Biological evaluation

Indigo naturalis has clear antiviral activity, as dose Radix Isatidis [13]. Neuraminidase (NA) is a necessary enzyme for replication of the influenza virus. At present, blocking the function of neuraminidase is recognized as an effective way to treat influenza presently. Oseltamivir and zanamivir are commonly used neuraminidase inhibitors, which commonly used to prevent and treat influenza. Therefore, our research team developed a biological assay to characterize the antiviral activity of indigo naturalis by determining the inhibitory rate of neuraminidase activity [14, 15]. The neuraminidase-based bioassay was high-throughput and simple, and could be used for evaluation and quality control of indigo naturalis.

Different from other traditional Chinese medicine, indigo naturalis has the characteristics of diverse plant sources and complex manufacturing technology. The quality of indigo naturalis can be affected by plant source, ratio of plant raw materials to lime, soaking time, climate and production season, which makes it difficult to standardize and standardize the manufacturing of indigo naturalis. At present, researches on the quality control of indigo naturalis are mainly focused on the organic compounds such as indigo, indirubin and tryptamine. However, lime is an essential raw material in the process of indigo naturalis manufacture. There are few studies on the problem of adding excessive lime. Shi et al. [16] found that many commercial indigo naturalis is a qualified product according to the standard, but most of them have high the total ash content and strong alkaline due to the addition of excessive lime. Obviously, excessive lime will affect the quality of indigo naturalis, and even affect the clinical efficacy and safety. Therefore, researchers should also focus on the control of inorganic content in indigo naturalis, except for the control of organic matter content.

## Traditional applications

It is easy to see from the name of indigo naturalis that this plant originated in India. The earliest literature records that indigo naturalis is abundant in Caoguo (now located in Afghanistan) and Turan (now located in Kazakhstan). Persians use indigo to dye their hair, and Chinese women use it to paint their eyebrows. In *Natural History*, Pliny mentions that indigo naturalis from the East was used by ancient Romans as paint, applied around the eyes as a cosmetic, or used as medicine, to promote wound healing. Since the Tang Dynasty, indigo naturalis has been used as a drug with remarkable therapeutic effects on fever, hemoptysis, convulsions in children, oral ulcers and sore throat. The earliest medical records in China appeared in the *Theory of Medicinal Properties* (Tang Dynasty, 627 A.D.). Later, it was included in many other famous medicinal books, including *Kaibao Materia Medica* (Song Dynasty, 973 A.D.), *Materia Medica Yanyi* (Song Dynasty, 1116 A.D.), and *Compendium of Materia Medica* (Ming Dynasty, 1590 A.D.). Indigo naturalis is indicated for various kinds of bleeding syndromes, such as hemoptysis and epistaxis, and its powder can be dipped with sterilized cotton and stuffed into the nasal cavity to stop bleeding. Indigo naturalis also can be used to cure oral diseases, such as a swollen, sore throat, ulcers, swelling, pain, and rottenness of mouth and tongue. In *Supplements to Commentarieson the Synopsis of the Golden Chamber*, it is used in a powder that is blown into the infected region. In addition to its use as a topical drug, indigo naturalis has been used in the past to treat convulsions and epilepsy in children. To increase its clinical effect, indigo naturalis is usually used in combination with other Chinese herbs, such as Gypsum Fibrosum, Bezoar, Snakegourd Seed and Radix Bupleuri.

## Pharmacology and mechanisms

### Antioxidation

Excessive oxygen free radicals can cause aging, inflammation and other diseases, which affect human health. Some components of medicinal plants have natural antioxidant effect, which is important in preventing and treating diseases caused by abnormal increase of oxygen free radicals [17]. It has been shown that indigo naturalis can play an antioxidant role by scavenging oxygen free radicals, improving the activity of superoxide dismutase, inhibiting the production of malondialdehyde, and stabilizing cell membrane permeability [18]. Lin [19] found that the extract of indigo naturalis could inhibit the increase of reactive oxygen species (ROS), H_2_O_2_ or derived hydroxyl radicals induced by exogenous H_2_O_2_. Meanwhile, it could interfere with protein modification induced by a lipid peroxidation product 4-hydroxy-2-nonenal (HNE), and protect keratinocytes from the electrophilic attack of HNE. Zhao [20] found that indigo and indirubin, the main components of indigo naturalis, could effectively scavenge superoxide anion. The mechanism may be associated with some electrophilic groups, such as ketones or aldehydes. These electrophilic groups could promote the release of hydrogen ion from hydroxyl groups and form non free radical superoxide anion, thus stabilizing superoxide anion. Another study found that indigo and indirubin could promote clearing of ROS, reduce the malondialdehyde content, promote the balance between the antioxidant system and ROS, inhibit lipid peroxidation, and protect cell membranes from oxidative damage [21]. Indigo can protect gastric mucosa from ethanol-induced gastric injury by preventing ethanol-induced DNA damage through non-enzymatic antioxidation and inhibition of polynuclear neutrophil infiltration [22]. What’s more, indigo naturalis can reduce the release of proinflammatory factors TNF-*α* and IL-6, indirectly reduce the level of ROS, and play the role of oxygen oxidation [23]. These results indicate that indigo naturalis and its some main components have potential as antioxidants.

### **Anti-inflammatory effects**

Inflammation, characterized by redness, swelling, heat and pain, is a body’s defense response to stimulation. However, excessive or long-term inflammatory response can lead to tissue damage [24]. Indigo naturalis and its some active ingredients have been proved to have significant anti-inflammatory effects, whose mechanism is the down-regulation of some inflammation factors and the inhibition of NO production [25, 26]. For instance, indigo naturalis and its main compound indigo can reduce the expression of TNF-*α*, IL-1, IL-6, IL-10, IL-22 and other inflammatory factors in plasma of UC mice via antagonizing TLRs/NF-*κ*B pathway and activating AhR pathway, thus improving inflammation [27, 28]. Indigo naturalis also can improve the oxidative stress level of colon tissue by increasing superoxide dismutase (SOD) and decreasing malondialdehyde (MDA) level, so as to control and reduce inflammatory reaction [29]. Indirubin inhibits the production of inflammatory cytokines through a variety of signaling pathways, and has a certain dose-dependent [30, 31]. A study found that indirubin significantly inhibited TLR4 and NF-*κ*B signaling and decreased the level of inflammatory cytokines in mice with lipopolysaccharide (LPS)-induced acute lung injury [25]. Except for the NF-*κ*B signaling pathway, indirubin also inhibits the MAPK signaling by decreasing the phosphorylation of extracellular ERK, P38, and JNK in LPS-induced mouse mastitis [32]. Another study found that indirubin can also reduce crypt deformation and mucosal damage, and reduce the infiltration of inflammatory cell in colonic mucosa [33]. Additionally, indigo naturalis can also play an anti-inflammatory role by inhibiting intracellular Ca^2+^ concentration. In formyl-L-methionyl-L-leucyl-L-phenylalanine (FMLP)-activated human neutrophils, indigo naturalis could significantly reduce O2(.-) generation and elastase release, inhibit calcium mobilization and reduce phosphorylation of extracellular p38 MAPK, JNK and some regulated kinase [34]. While indigo naturalis not affect cellular cAMP levels. Interestingly, neither indirubin, indigo nor tryptanthrin had similar effects in FMLP-activated human neutrophils and this result indicates that there are some unknown activities. Although indigo naturalis has significant anti-inflammatory effect, its anti-inflammatory mechanism and active ingredients need to be further explored.

### **Antibacterial**

Indigo naturalis and its active components have good antibacterial, antifungal and antiviral activities [35–37]. The antimicrobial assays in *vitro* suggested that the ethyl acetate extract of indigo naturalis could remarkably inhibit gram-positive bacteria (methicillin-resistant *Staphylococcus aureus, Staphylococcus epidermidis*, and *Staphylococcus aureus*) and mildly inhibit noncutaneous fungal pathogens of onychomycosis (*Aspergillus fumigatus* and *Candida albicans*) [38]. For these bacteria, tryptanthrin has better antibacterial activity than indigo. It could suppress the growth of pathogens by intercalating into bacterial DNA [39]. Indigo can cause extensive oxidative damage so as to host cell death by promoting superoxide production [40]. In the absence of light, indigo can have antibacterial effects on *Staphylococcus aureus*, *Staphylococcus epidermidis*, *Escherichia coli*, *Proteus vulgaris*, and *Candida albicans* (minimum inhibitory concentration [MIC] = 240 µm). Interestingly, in the presence of light, a lower dose of indigo (MIC/2) can show antibacterial effects [40]. Indirubin can also inhibit *Staphylococcus aureus* and enhance the activity of ciprofloxacin. This mechanism may be interrelated to the inhibition of NorA efflux pump in *Staphylococcus aureus* [41]. Indigo is the isomer of indirubin, but there is no related research on its inhibition of NorA efflux pump.

### Antiviral

Indole alkaloids, especially bisindole nuclear alkaloids, have been widely concerned in the field of anti-infection due to their special structure, strong analogues, high selectivity and low side effects [42]. Indigo naturalis is rich in indole alkaloids, and the anti-virus research of indigo naturalis mainly focuses on indigo and indirubin. Influenza virus is the pathogen of influenza, especially influenza A virus is easy to mutate, causing a worldwide pandemic [43]. Indirubin could reduce activation of the chemokine regulated on, the expression of normal T cell and the secretion of influenza virus A/NWS/33- infected H292 human epithelial cell line. And this inhibitory effect was also observed as well as influenza virus B/Lee-infected cells [44]. In *vivo* test, Indirubin can reduce the sensitivity of stress-induced mice to influenza virus (H1N1), thereby reducing lung injury and improving survival rate [36]. Further study showed that indirubin could promote the formation of IFN- glycolic acid and increase the expression of IFN-*β* and IFN-induced transmembrane 3 through mitochondrial antiviral signal copper aluminum, thus maintaining the morphology and function of mitochondria after H1N1 infection. Indigo and indirubin have strong killing activity against Japanese encephalitis virus, which may be related to block virus attachment. Especially indirubin has a strong protective effect on lethal Japanese encephalitis virus model [45]. In addition, indigo can inhibit the 3C-like protease (3CL^pro^) of SARS-coronavirus in dose dependence, which can induce the hydrolysis of replication enzyme polypeptides 1a and 1ab into functional proteins [45]. The above studies indicate that indigo naturalis and its active ingredients are expected to become potential antiviral drugs, especially drug-resistant viruses. Meanwhile, more in-depth experiments are needed to prove their potential as antiviral drugs.

### Immune modulation

Indigo naturalis, as traditional Chinese medicine, has been widely used in autoimmune diseases and inflammatory diseases, such as psoriasis and allergic contact dermatitis [46, 47]. A study has shown that indigo naturalis can inhibit oxazolidone induced allergic dermatitis by reducing eosinophil recruitment and production of Th2 related cytokines. But at the same time, indigo naturalis can change the structure of intestinal flora and aggravate colitis [48]. Indirubin can decrease serum IgE and cytokines production, and normalize the expression of NF-*κ*B, I*κ*B-*α* and MAP kinase expression in a dose dependence by regulating the co-expression of Th1 and Th2-mediated immune response [47]. Indirubin can also improve the proliferation of keratinocytes, inhibit the activation of JAK3/STAT3 pathway, reduce the osmosis of CD3 + T cells, *γδ*T cells and CD11b + neutrophils, and decrease the level of *γδ*T cells in spleen and lymph nodes [49]. What’s more, indirubin can selectively increase the number of CD4 + CD25 + Treg cells, which is important in the induction and maintenance of peripheral self-tolerance, leading to the induction of immune tolerance [50]. Man et al [51]. detected the effect of indirubin on ATP-induced macrophage immune response: neutral red dye uptake was used to measure cell death, and the ROS level was tested by dihydroethidium fluorescence probe. The results showed that indirubin could reverse the ATP-induced decrease in phagocytosis and cell death, and inhibit ATP-induced ROS production, indicating that indirubin blocked ATP-induced macrophage immune response.

### Antitumor

Malignant tumor has become one of the major diseases threatening human beings. It is urgent to find drugs with high selectivity, small side effects and strong curative effect. Among numerous pharmacological actions of indigo naturalis, the antitumor effect is of great interest. Indirubin is of importance in the antitumor effects of indigo naturalis. In recent years, indirubin in indigo naturalis has attracted more and more attention in the field of anti-tumor. It has significant antiproliferative activity and is a powerful apoptosis inducer for a variety of tumor cells, such as cervical cancer, liver cancer and lymphoma cell lines [52, 53]. Realgar-indigo naturalis, a traditional Chinese medicine preparation composed of indigo naturalis, *Salvia miltiorrhiza* and realgar, was used to treat human acute promyelocytic leukemia (APL). The complete remission rate of APL patients with Realgar-indigo naturalis was 96.7–98%, and the 5-year overall survival rate was 86.88% [54, 55]. Wang et al. [56] observed the effects of tetrasulfide (A) in realgar, indirubin (I) in indigo naturalis and tanshinone IIA (T) in *Salvia miltiorrhiza* in vivo and in vitro. They found that compared with single component or dual drug combination, ATI can increase the ubiquitination/degradation of promyelocytic leukemia (PML)-retinoic acid receptor *α* (RAR*α*) oncoprotein, enhance the reprogramming of myeloid differentiation regulators, and enhance G_1_/G_0_ block in APL cells by striking multiple targets. In addition, ATI enhanced the level of Aquaglycoporin 9, promoted the transport of a to APL cells, and then promoted the degradation and therapeutic effect of PML-RARα mediated by A. In the cell viability and induce apoptosis test of 2 human ovarian cancer cell lines, indirubin can inhibit cell activity and induce apoptosis with dose dependence by downregulating the phosphorylation of STAT3 (tyr705), and significantly suppressing the expression of cyclin D1 and c-myc downstream of STAT3 [31]. Indirubin can inhibit tumor necrosis factor induced NF-*κ*B activation in a dose and time-dependence. It can also inhibit the expression of NF-*κ*B regulated genes, including anti apoptotic genes (iap1, iap2, Bcl-2, BCL XL and TRAF1), proliferation genes (cyclin D1 and c-myc), and invasive genes (COX-2 and MMP-9), thus playing anti-inflammatory and anti-tumor activities [30]. Indirubin can not only inhibit the proliferation of cancer cells, but also the angiogenesis of tumor growth process. A study found that indirubin suppressed JAK/STAT3 signaling pathway to inhibit tumor angiogenesis [57] .

Tryptanthrin in indigo naturalis also has antitumor activity. Tryptanthrin can make the murine myeloid leukemia WEHI-3B JCS cells stay in G_0_/G_1_ phase, and down-regulate the expression of cyclin D2, D3, CDK2, 4, 6 genes, so as to inhibit the proliferation of WEHI-3B JCS and play an anti-tumor role [58]. In non-melanoma skin cancer, tryptanthrin can inhibit the proliferation of hair follicle cells by inhibiting the activation of ERK_1/2_ and p38 and reducing the expression of *β*-Catenin [59]. Tryptanthrin can also inhibit angiogenesis by regulating VEGFR2 and ERK_1/2_ signaling pathways and reducing the level of Ang-1, PDGFB and MMP2 [60]. Additionally, tryptanthrin can be used as a potential chemotherapy adjuvant to inverse multidrug resistance in a breast cancer cell line MCF-7 by down-regulating multidrug resistance gene 1 [61].

### Others

Indirubin in indigo naturalis can inhibit wnt/β-Catenin signal transduction by restoring the expression of wif-1, inhibit the expression of TGase1 and down-regulate CDK1 to inhibit the proliferation of keratinocytes [62–64]. Tryptophan, another active component of indigo naturalis, has been proved to have the ability to inhibit keratinocytes, which is stronger than indigo and indirubin [65, 66]. Tryptophan can also inhibit the activity of apelin promoter, and inhibit the proliferation and migration of vascular endothelial cells by causing G_2_/M phase arrest, inhibiting Akt and FAK pathway, thus achieving anti angiogenesis effect [67]. Other studies found that indigo naturalis could repair intestinal mucosa. It can increase the expression of vascular endothelial growth factor, epidermal growth factor, and occludin protein, and promote the repair of intestinal mucosa [68]. Except for promoting intestinal mucosal repairing and ulcer healing, indigo naturalis also can inhibit the gene expression of MMP-1 and TIMP-1 and reduce the damage of colonic mucosa by inhibiting Smads signaling pathway and activating MAPK signal pathway [69, 70]. Intestinal microflora is a hot spot in the treatment of ulcerative colitis in recent years. Indigo naturalis was found that it could reduce the relative abundance of harmful bacteria in the colon, increase the relative abundance of probiotics, and maintain the intestinal microbial homeostasis [71].

## Clinical applications

### Acute promyelocytic leukemia

APL is a common malignant disease in the blood system and characterized by abnormal coagulation function and acute promyelocytosis [72]. All trans retinoic acid (ATRA) and arsenic therapies are the most widely used therapies for APL, which has serious adverse reactions, high toxicity and relapse rate [73]. Indigo naturalis alone has no effect on APL, but indirubin in indigo naturalis could enhance the inhibition of arsenic disulfide in realgar on the proliferation and apoptosis of diffuse large celllymphoma cells and promote the transport of arsenic disulfide to APL cells, thus enhancing the degradation and treatment effect of PML-RAR α mediated by arsenic disulfide and accelerating the apoptosis of NB4 cells [56, 74]. Clinical studies showed that the relapse rate of APL was 12.90% and the total mortality rate was 6.45% by alternately using chemotherapy and realgar-indigo naturalis formula (RIF), mainly consist in realgar and indigo naturalis. Compared with ATRA therapy, there was no significant difference in blood parameters [8, 75]. It should be noted that patients with RIF may have adverse reactions such as nausea, vomiting, diarrhea, abdominal pain and rash, but there are no serious adverse reactions [76]. In non-high-risk APL clinical trial, the results showed that oral RIF plus ATRA has the same efficacy as intravenous arsenic trioxide plus ATRA and can replace the latter [77]. The adverse reactions of the two therapies were compared. It was found that liver aspartate aminotransferase or alanine aminotransferase concentration increased in 9% patients with RIF plus ATRA and 6% patients with arsenic trioxide plus ATRA, and grade 2–3 infection occurred in 14% patients with RIF plus ATRA and 23% patients with arsenic trioxide plus ATRA. In the arsenic trioxide-ATRA group, there are two patients died during induction therapy. In children with APL, the 5-year event-free survival of RIF group is 100%, as well as intravenous arsenic trioxide after an average follow-up of 3 years. The adverse reactions were mild, but the hospitalization time of RIF was significantly lower than that of intravenous arsenic trioxide [78]. The above studies show that indigo naturalis is a potential adjuvant drug for treating APL.

### Psoriasis

Psoriasis is a chronic immune related skin disease. It is characterized by erythema, scales and pruritus, which is determined by polygenic inheritance and induced by multiple environmental factors [79]. The clinical observation of indigo naturalis in treating psoriasis has been reported in the 1980 s, and the overall cure rate is 69.6% [80]. According to the modern research results, indigo naturalis mainly treats psoriasis by inhibiting the excessive proliferation of keratinocytes, regulating the body immunity, reducing inflammation and anti angiogenes [62–65, 67]. At present, indigo naturalis is mostly used as external preparation in treating psoriasis vulgaris and nail psoriasis (Table 1). It should be noted that in a clinical report, 4 out of 42 subjects had pruritus after using indigo naturalis ointment [81]. The safety of indigo naturalis for external use needs further evaluation. Moreover, the indigo naturalis ointment are prepared by the hospital itself without no unified standard. Therefore, it is necessary to develop indigo naturalis into qualified patent medicine as soon as possible.

**Table 1:** Clinical experiment of treating psoriasis with indigo naturalis

Drugs	Clinic types of experiments	Types of psoriasis	Number of examinees	Usage time	Results	Refs.
Indigo naturalis oil extract	A non-controlled pilot study	Nail psoriasis	n = 28	24 weeks	The Nail Psoriasis Severity Index (NAPSI) decreased from 36.1 ± 14.7 to 14.9 ± 11.1, and the average Nasi score decreased from 11.7 ± 3.9 to 3.6 ± 3.2	[82]
Indigo naturalis ointment	A randomized, double-blind, placebo-controlled study	Moderate psoriasis	n = 24 (drug:16, placebo: 8)	8 weeks	In 56.3% of patients, the psoriasis area and severity index scores were improved by 75%, and IL-17 in skin genes of these patients was significantly down regulated compared with placebo	[83]
Indigo naturalis ointment or vehicle ointment	A randomized, observer-blind, vehicle-controlled study	Recalcitrant psoriasis	n = 42	12 weeks	Compared with placebo, the scores of erythema, scale and lump and plaque area of indigo naturalis in treatment site were significantly reduced. The symptoms of psoriasis disappeared or nearly disappeared in 74% of patients with indigo naturalis	[81]
Indigo naturalis composite ointment	–	Pediatric psoriasis	8-year-old boy	8 weeks	After treating with indigo naturalis composite ointment, the lesions of systemic psoriasis disappeared completely, and the affected body surface decreased from nearly 80–0%. Remission has lasted for over 2 years without any adverse reaction	[84]
Indigo naturalis oil extract drops	–	Pediatric nail psoriasis with periodic pustular eruption	A 13-year-old girl	6 months	After treat in one month, the pustulation of the nails and the crusted, keratotic erythematous lesions disappeared. A completely normal nail unit without Beau’s lines was achieved after 6 months without any adverse reaction Remission has lasted for over 1 year	[85]

### Ulcerative colitis (UC)

UC is a common clinical digestive tract disease, mainly manifested as abdominal pain, diarrhea and bloody stool, and intestinal mucosal ulcer, erosion, bleeding and so on can be seen under the microscope [86]. According to relevant reports, indigo naturalis mainly treats ulcerative colitis by reducing inflammation, repairing intestinal mucosa and regulating intestinal flora [33, 69–71]. According to clinical observation results of indigo naturalis, it has curative effect in the treatment of UC. At the same time, there are also adverse reactions such as gastrointestinal reactions, liver injury and pulmonary hypertension, but there is no death (Table 2). In order to further research the safety of indigo naturalis in treating UC, a follow-up survey was carried out in 877 patients using indigo naturalis. It was found that 91 patients (107 cases) had adverse reactions, including 40 cases of liver dysfunction, 21 cases of gastrointestinal symptoms, 13 cases of headache and 11 cases of pulmonary hypertension. Liver dysfunction is mildly reversible. Surgery is required in 40% of patients with intestinal adhesions. After stopping the treatment, pulmonary hypertension could heal itself without any death [87]. Although indigo naturalis has some side effects in the treatment of UC, it is still a potential drug. Meanwhile, it is necessary to monitor adverse reactions of indigo naturalis for a long-time use.

**Table 2:** Clinical experiment of indigo naturalis in treating UC

Drugs	Clinic types of experiments	Number of examinees	Usage time	Results	Adverse reactions	Refs.
Indigo naturalis	Retrospective observational study	n = 14	8 weeks	In 10 active UC patients 50% had clinical response and 40% achieved clinical remission. Rachmilewitz endoscopic index (REI), and UC endoscopy index of severity (UCEIS). the Mayo endoscopic subscore (MES) decreased from 2 (2–3) to 1 (1–2), the Rachmilewitz endoscopic index (REI) decreased from 7 (5.5–11) to 3 (1–7), and the UC endoscopy index (UCEIS) of severity decreased from 3 (3-4.5) to 1 (0.5–3.5)	One patient developed right colitis with intestinal wall thickening and edema	[88]
Indigo naturalis oil suppository	Open-label, single-center, prospective pilot study	n = 10	4 weeks	30% of patients achieved clinical remission, 40% of patients had mucosal healing. Mucosal healing was observed in 80% of patients with MES in the Rectum (R-MES) score of 2, but not in patients with scores of 3	One patient developed perianal pain	[89]
Indigo naturalis	A multicenter, double-blind trial	n = 86	8 weeks	The clinical response of UC was correlated with the oral dose of indigo naturalis. 13.6% of patients had clinical response to placebo; 69.6% to 0.5 g indigo naturalis; 75.0% to 1.0 g indigo naturalis and 81.0% to 2.0 g indigo naturalis. At week 8, 56.6% patients with 0.5 g, 60% with 1.0 g and 47.6% with 2.0 g indigo naturalis received clinical relief compared to 13.6% placebo	Mild liver dysfunction was observed in 10 patients taking indigo naturalis, but there were no significant adverse reactions. Unfortunately, the experiment was terminated because a patient took indigo naturalis by heself and developed pulmonary hypertension	[90]
Indigo naturalis	Retrospective observational study	n = 17 in UC and n = 8 in Crohn’s disease (CD)	8 weeks	There are 94.1% and 88.2% in UC and 37.5% and 25.0% in CD receiving Clinical response and clinical remission, respectively	During follow-up, 10 patients developed adverse reactions, and 3 patients experienced severe adverse reactions, including 2 cases of acute colitis requiring hospitalization and 1 case of acute colitis intussusception requiring surgical treatment	[91]
			52 weeks And 104 weeks	Through non-responders imputation analyses at weeks 52 and 104, the clinical remission rates were 76.4% and 70.4% in UC patients and 25.0% and 25.0% in CD patients		

### Other clinical applications

In addition to the treatment of leukemia, psoriasis and colitis, modern research has found that indigo naturalis can also be used to treat liver injury, nephritis, mouth ulcer and other skin diseases, which may be related to its anti-inflammatory, antioxidant and immune regulatory effects [18, 20, 92, 93]. However, research of these diseases mostly stays in the basic stage, and more data support is needed from the clinical use.

### Application challenges and solutions

In recent years, indigo naturalis has been widely used in the treatment of leukemia, psoriasis, ulcerative colitis and other diseases, as well as been recognized by experimental research and clinical observation. However, it seems that indigo naturalis lacks accurate dose-response relationship research and direct clinical big data to guide clinical application, especially its safety. Therefore, it is necessary to carry out clinical observations with randomized controlled, double-blind, double-dummy, multi-center and large sample, which is conducive to the further development and clinical application of indigo naturalis. Additionally, it should speed up the development of indigo naturalis preparation to be convenient for the clinical application and improve the safety of indigo naturalis.

## Conclusion

As a national medicine with a long history, indigo naturalis has a variety of biological activities and broad application prospects. Although the anti-inflammatory and anti-tumor effects of indigo naturalis have been widely recognized by the medical community, there are some adverse reactions such as gastrointestinal reactions, skin itching, reversible liver dysfunction and so on, which can not be ignored. The advantages and disadvantages should be fully considered in the use of indigo naturalis. Additionally, researches of indigo naturalis mainly focused on indigo, indirubin and tryptophan, but less on other components. It is necessary to use modern pharmaceutical research methods to study the efficacy and mechanism of its effective components. Meanwhile, it can also be used for drug design of indigo naturalis and its active ingredients to reduce adverse reactions, which makes indigo naturalis safer in clinical application.

## Data Availability

The datasets used in this study are available from the corresponding author upon reasonable request.

## Abbreviations

ROS: Reactive oxygen species
HNE: Lipid peroxidation product 4-hydroxy-2-nonenal
LPS: Lipopolysaccharide
FMLP: Formyl-L-methionyl-L-leucyl-L-phenylalanine
APL: Acute promyelocytic leukemia
PML: Promyelocytic leukemia
RAR*α*: Retinoic acid receptor*α*
ATRA: All trans retinoic acid
RIF: Realgar-Indigo naturalis formula
UC: Ulcerative colitis
